# Weight management interventions in adults with intellectual disabilities and obesity: a systematic review of the evidence

**DOI:** 10.1186/1475-2891-12-132

**Published:** 2013-09-23

**Authors:** Dimitrios Spanos, Craig Andrew Melville, Catherine Ruth Hankey

**Affiliations:** 1 College of Medical, Veterinary and Medical and Life Sciences, University of Glasgow, Glasgow G31 2ER, UK; 2 Learning Disabilities Psychiatry, College of Medical Veterinary and Life Sciences, Institute of Mental Health & Wellbeing, University of Glasgow, Glasgow G12 0XH, UK; 3 Human Nutrition, College of Medical, Veterinary and Medical and Life Sciences, University of Glasgow, Glasgow Royal Infirmary, Level 2, New Lister Building, Glasgow G31 2ER, UK

**Keywords:** Intellectual disabilities, Obesity, Weight loss

## Abstract

To evaluate the clinical effectiveness of weight management interventions in adults with intellectual disabilities (ID) and obesity using recommendations from current clinical guidelines for the first line management of obesity in adults. Full papers on lifestyle modification interventions published between 1982 to 2011 were sought by searching the Medline, Embase, PsycINFO and CINAHL databases. Studies were evaluated based on 1) intervention components, 2) methodology, 3) attrition rate 4) reported weight loss and 5) duration of follow up. Twenty two studies met the inclusion criteria. The interventions were classified according to inclusion of the following components: behaviour change alone, behaviour change plus physical activity, dietary advice or physical activity alone, dietary plus physical activity advice and multi-component (all three components). The majority of the studies had the same methodological limitations: no sample size justification, small heterogeneous samples, no information on randomisation methodologies. Eight studies were classified as multi-component interventions, of which one study used a 600 kilocalorie (2510 kilojoule) daily energy deficit diet. Study durations were mostly below the duration recommended in clinical guidelines and varied widely. No study included an exercise program promoting 225–300 minutes or more of moderate intensity physical activity per week but the majority of the studies used the same behaviour change techniques. Three studies reported clinically significant weight loss (≥ 5%) at six months post intervention. Current data indicate weight management interventions in those with ID differ from recommended practice and further studies to examine the effectiveness of multi-component weight management interventions for adults with ID and obesity are justified.

## Introduction

Intellectual disability is defined as the “disability characterized by significant limitations both in intellectual functioning and in adaptive behaviour, which covers many everyday social and practical skills” [1]. Obesity is an important health issue for adults with ID with an estimated prevalence equal to 27% in UK and 33.6% in USA [2]. However, there appears to be only a limited evidence-base underpinning the management of obesity in this population group [3]. Previous reviews of weight loss interventions in adults with ID found that studies have important methodological weaknesses including small and often unjustified sample sizes, heterogeneous samples and non-randomised designs [3-6]. However, no review to date has examined the effectiveness of these studies against criteria used in international clinical guidelines for the first line management of obesity [7-9]. These are:

1. Adults who are overweight or obese should aim for a clinically significant 5-10% weight loss (approximately 5–10 kilograms (kg)) from initial body weight for three to six months.

2. Multi-component lifestyle interventions, that include:

•Dietary advice to incorporate a diet with 600 kilocalorie (kcal) (2510 kilojoule (kJ)) per day to 1000 kcal (4186 kJ) per day deficit or low energy content by lowering fat intake

•Physical activity should be increased to 225-300 min or more of moderate intensity physical activity per week

•Behaviour change strategies to facilitate the dietary and activity changes advocated

3. After six months of weight management adults should be encouraged to develop skills relevant to maintaining weight losses.

Therefore, this review aims to answer the following research questions:

•What components are included in weight loss interventions for adults with ID?

•Are weight loss interventions for adults with ID associated with a clinically significant weight loss (5-10% or 5-10 kg weight loss from initial body weight)?

•Do interventions include a weight loss maintenance component?

## Method

### Systematic electronic database searching

The present study comprised an electronic search of four electronic databases for the years 1982–2011: Medline, Embase, PsycINFO and CINAHL. Search terms included ID, mental retardation, learning disorders, mentally disabled persons, developmental disabilities, obese, overweight, weight gain, weight loss, body mass index (BMI), diet, low fat diet, low calorie diet, diet restriction, behaviour therapy, cognitive therapy, family therapy, lifestyle, exercise, physical activity, physical education, nutrition education, health promotion, health education. Articles were selected on the basis of the presence of these terms in the title and abstract.

### Selection criteria

The selection of studies for this review was not restricted to finding randomised controlled studies (RCT) design, but included according to the following eligibility criteria:

•Valid diagnosis of ID at study enrollment

•Adults (≥ 18 years of age)

•Record of weight status (e.g. obese, overweight) based on the diagnostic criteria valid at the time of study

•Non-surgical or pharmacological interventions

•Impact of intervention on total body weight and/or BMI

### Exclusion criteria

**Figure 1:**
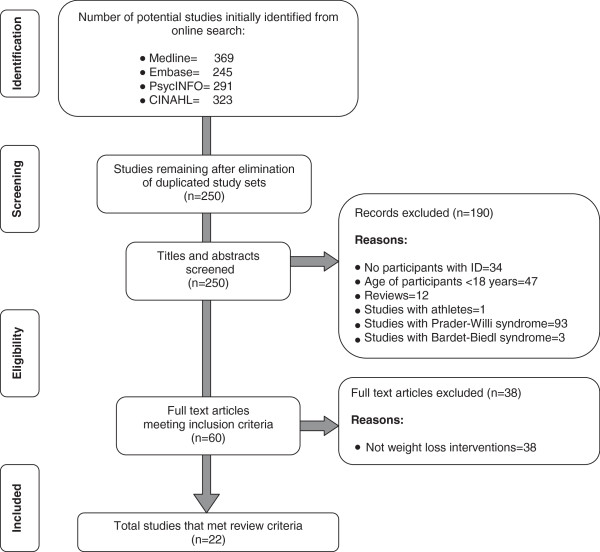
Process of selection of studies for inclusion in the review.

Studies on pharmacotherapy and surgery were excluded. Studies that investigated weight management in adults were obesity is attributed to specific genetic syndromes such as Prader-Willi syndrome, Cohen syndrome or Bardet-Biedl syndrome were also excluded. Studies that included Special Olympics athletes were also excluded. The process of selection of studies for inclusion in the review can be seen in Figure 1.

### Data extraction

A standardised data extraction form was developed for this review. The data were extracted by one researcher (DS) and then discussed and reviewed by a second researcher (CM). Details of each study were extracted regarding:

•author, title, year of publication

•research question, study design, duration, method of randomization, blinding, length of follow up

•sample characteristics, power calculation, sample size, diagnostic criteria for level of ID and weight status, attrition

•outcome measures: weight and BMI change

•intervention components

•results

•conclusion.

### Evaluation of studies

Evaluation of quality and results data was undertaken by one reviewer (DS). The findings and points for clarification were discussed with two reviewers with extensive experience of the clinical area and review methodology (CM, CRH). For the assessment of the quality of the studies and interventions a checklist was developed based on the criteria of the Centre for Reviews and Dissemination (CRD) (University of York) [10], and the PRISMA checklist [11]. Since the review was likely to include a diverse range of studies it was preferable to consider individual aspects of methodological quality in the quality assessment and synthesis [10]. Some of the criteria included in the assessment checklist were the following:

•The presence of sampling bias e.g. power calculation, heterogeneity

•Detailed description of the methodology of randomization

•Replicability based on detailed description of intervention

•The level and explanation for attrition

•Follow up measurements

Each study was evaluated using the key recommendations of national and international clinical guidelines for the management of obesity in adults [7-9]. Therefore, studies were assessed based on the components of each intervention and the study outcomes e.g. report of clinically significant weight loss.

## Results

### Literature search

Twenty two studies that reported the effectiveness of specific interventions designed to achieve weight loss in adults with ID and obesity met the inclusion criteria and were included in this systematic review (total number of articles identified and the total number selected for inclusion in the review can be seen in Figure 1).

The majority of the studies took place in the USA (n = 12), four studies took place in the UK and the rest in Hungary, Italy, South Africa, Portugal, Taiwan and Australia. Most of the studies were classified as uncontrolled or single stranded studies where before and after measurements were made. According to Grimshaw et al. [12] quasi-experimental studies often are conducted where there are practical and ethical barriers to conducting randomized controlled trials. In this review when participants were randomly assigned the groups were referred as control groups and when not randomly assigned as comparison groups.

Based on the description of each component and specific definitions of dietary interventions [9] on behaviour change techniques [13] and multi-component interventions [14] the interventions were classified as:

1. Behaviour change

2. Behaviour change plus physical activity

3. Dietary

4. Physical activity

5. Diet plus physical activity

6. Multi-component (three or more components)

The components of the interventions, the study outcomes and limitations are described in the text. Further details are given in separate tables one to six. The tables report results for mean weight or BMI change, where possible. Weight change in text is reported as absolute weight loss or weight gain, as variation of weight change was only sometimes reported in papers.

### Behaviour change interventions

Single component psychological interventions used behaviour change to provide the participants “with coping skills to handle cues to overeat and manage lapses in the diet and physical activity when they occur” [13] (see Table 1).

**Table 1:** Behaviour change interventions

**Study/ Location/ Type**	**Participants**	**Intervention**	**Follow up**	**Results**
Fox 1985 [15] USA Community Quasi-experimental with a comparison group	**(a) Parent involvement group** n = 8 Weight status: all obese Gender: 4 females, 4 males Age (years)^a^: 27(2.7) ID: moderate	**Duration:** 10 week of 2 group sessions per week (60 min each).**(a)** Behaviour change methods based on Rotatori [18]. Parents involved with intervention strategies e.g. homework and reward systems.	10 week	**(a)** Mean weight change, kg: - 3.4 (range: -1.81 to -4.9) **(b)** Mean weight change, kg: -1.09 (range: +2.27 to -3.63). Significant between group difference (*p* < 0.05)
	**(b) Subject involvement** n = 7 Weight status: all obese Gender: all malesAge (years)^a^: 29 (2.2) ID: moderateAttrition/drop out: none	**(b)** Same as (a) but parents were not involved. **Maintenance:** 3 monthly meetings reviewing eating and activity behaviours, reward system continued.	3 month	**(a)** Mean weight change, kg: + 0.7 (range: +3.6 to -2.7) **(b)** Mean weight change, kg: +0.1 (range: +3.2 to -5.4)No significant between group difference
			6 month	**(a)** Mean weight gain, kg: +1.8 (range: -3.2 to +5.4) **(b)** Mean weight gain, kg: +2.8 (range: 0 to +6.8) No significant between group difference
McCarran 1990 [16] USA Community Quasi-experimental with a comparison group	Total n = 12, 8 completers Weight status: 22-109% overweight Gender: 7 females, 1 male Age (years): 19-42 ID: cerebral palsy, IQ: 50-80 Attrition/drop out: 4 drop outs	**Duration:** 14 weeks of 3 group sessions per week (60 min each) led by a graduate student and undergraduate.**(a) Home Help group:** Behaviour change methods based on Rotatori [18]. Frequent contacts with the parents/caretakers by the group leader.	14 weeks	**(a)** Mean weight change, kg:-2.5^b^**(b)** Mean weight change, kg: -1.2 Significant weight loss for both groups (*p* < 0.01) No significant between group difference Significant reduction in BMI, weight reduction quotient, % overweight for both groups (*p* < 0.05)
		**(b) No Help group:** Behaviour change methods same as (a) but with no communication with the parents/caretakers. **Maintenance:** 5 weeks of practicing techniques identified as problematic.	12 months	**(a)** Mean weight change, kg: -1.5 **(b)** Mean weight change, kg: +0.5
Sailer 2006 [17] USA Community Uncontrolled Quasi-experimental study	Total n = 6 Weight status: all obese Gender: 4 females, 2 males Age (years): 34-54 ID: mild Attrition/drop out: none	**Duration:** 10 weekly group sessions (60 min each) and phone call contacts. Behaviour change methods based on Rotatori [18]. **Maintenance:** none reported	10 week	Mean weight change, kg: -2.5 (range: +0.5 to -8.2)
			1 month	Mean weight change, kg: -1.5 (range: +2.26 to -5.9)

^a^data are mean values (SD).

^b^range not reported.

The duration of the interventions varied from 10 to 14 weeks and they were all delivered in group sessions in the community [15-17]. The intervention in only one study was delivered by an individual specialized in ID [16]. The remaining studies did not provide such information on the qualifications of those delivering the intervention.

### Intervention components

#### Behaviour change

Behaviour change interventions were based on the comprehensive behaviour self-control programme developed by Rotatori and Fox [18]. The intervention was accompanied by a specific manual [18] which aimed to change the eating habits, activity levels and self-reinforcement patterns of the participants by gradually introducing new behaviour change techniques. The process included seven main steps: 1) increase self-awareness of body weight, 2) control snacking frequency 3) control triggers that lead to overeating 4) adopt a healthy balanced diet 5) self-control of overeating 6) increase physical activity 7) consume low calorie foods [16].

Two key behaviour change techniques were the processes of self-monitoring and self-reinforcement. For this reason participants were asked to complete food diaries and reward themselves for achieving specific changes of their dietary habits. Approaches included: control triggers that lead to overeating, consumption of only one portion of a meal, reducing the rate of eating, limiting meal or snack consumption to one location in the home, reducing snacking frequency, putting the utensils down after each bite, not always consuming the complete meal and eating low calorie foods. Physical activity patterns were targeted by recommendation of simple changes in everyday activities (e.g. taking the stairs instead of the elevator). In addition, participants were given weekly homework assignments to ensure they practiced the learned techniques at home. Non-food reward strategies were used to support attendance at the sessions, and maintain encouragement and motivation to lose weight.

In a second study using the self-control programme parents had to assist the participants with daily homework and encourage them to practice what they have learned in relation to eating, activity and self-reinforcement [15]. Parents were also involved with a weekly reward procedure and were asked to offer a non-food reward for a weight loss. Another study involved carers by sending written material to them weekly [16].

Two studies incorporated a weight loss maintenance intervention [15,16]. Fox et al. [15] included a maintenance period comprising three meetings held monthly that focused on reviewing new eating and activity behaviours. However, the reward system was used to promote further weight loss during that phase. McCarran and Andrasik [16] followed the weight loss intervention with a five week weight maintenance phase. During these twice-weekly, 60-minute maintenance training meetings the researchers continued to practice the techniques identified as problematic for the participants and promoted strategies that could help the participants to maintain any weight losses.

### Study outcomes

None of these studies reported a mean clinically significant weight loss of either 5-10% or 5-10 kg of initial body weight. Although McCarran and Andrasik [16] reported a statistically significant weight reduction (p < 0.01) this was only equal to 2.5 kg at 14 weeks.

Fox et al. [15] reported a mean weight loss of 3.4 kg for the group with parent-involvement, which was significantly different from the group with no parent-involvement. However, McCarran and Andrasik [16] found greater but not significantly different weight loss for participants that had their carers involved than the participants who did not.

Post intervention weight loss was not sustained with Fox et al. [15] reporting weight regain at three and six months follow up.

### Study limitations

Limitations for the behaviour change intervention studies included no sample size justification, small sample size (ranging from six to 15) and no random allocation. The duration of follow up measurements was short, with one study McCarran and Andrasik [16] reporting a 12 month follow up. Attrition was low, with none for two studies [15,17]. McCarran and Andrasik [16] reported having incidents of drop outs (n = 4) due to scheduling conflicts (n = 3) and due to family related conflicts (n = 1).

### Behaviour change plus physical activity interventions

These studies were principally behaviour change based interventions that incorporated specific physical activity advice or a physical activity programme to support increased energy expenditure (see Table 2).

**Table 2:** Behaviour change plus physical activity

**Study/ Location/ Type**	**Participants**	**Intervention**	**Follow up**	**Results**
Fox 1984 [19] USA Community Quasi-experimental study with a control group	**(a) Behaviour Therapy group (BT) **n = 8 Weight status, % overweight^a^: 44.4 (35.4) Gender: 5 females, 3 males Age (years)^a^: 29.5 (7.2) ID, IQ ^a^: 42.1 (8.4)	**Duration:** 10 weeks of 2 group sessions per week (60 min each) led by a researcher and a recreational therapist. **(a)** Behaviour change methods based on Rotatori [18]. Parents involved with intervention strategies e.g. homework, reward systems. Phone contacts were also included. **Activity:** calisthenics and aerobic exercises (2 times a day) plus walking and using stairs.	10 week	**a)** Mean weight change, kg:- 3.3 ( range: +0.4 to + 7.26) % weight loss: 5.7 **(b)** Mean weight change, kg:-3.72 (range: +1.36 to +7.7) % weight loss:6.6
	**(b) BT + Buddy reinforcement** n = 8 Weight status, % overweight ^a^: 34.7 (18.3) Age, (years)^a^: 27.5 ( 5.4) ID, IQ ^a^: 46.3 (12.1) Attrition/drop out: none	**(b)** Same as (a) plus participants were paired into 4 buddy teams.**Maintenance:** 5 weekly meetings reviewing behaviour change strategies, applying reinforcement and reducing homework. Weight loss was still promoted.	15 week	**(a)** Mean weight change, kg:-0.9 (range: +0.4 to -2.72) **(b)** Mean weight change, kg:-1.04 (range:+0.98 to -3.2)
			52 weeks	**(a)** Mean weight change, kg: -0.27 (range: +2.25 to -3.6) from baseline
				**(b)** Mean weight change, kg: -1.8 (range: +2.7 to -14.8) Total 37.5 % maintained weight.No significant between–group difference at 10 week, 15 week and 52 weeks follow up
Fisher 1986 [20] USA Community Quasi-experimental study with a control group	Total n = 17 Weight status: All obese Gender: All femalesAge (years) ≈ 20 ID: mild to moderate Attrition/drop out: none	**Duration:** 8 week group sessions**(a) Behaviour self control group**: Behaviour change methods based on Rotatori [18].	8 week	**(a)** Mean weight change, kg: -1^b^
				**(b)** Mean weight change, kg: -0.6 No significant difference between (a) and (b)
		**(b) Behaviour self control group plus physical activity:** Behaviour change methods same as (a). **Activity:** walking (10 min/day increased to 30 min by week 8) **Maintenance:** none reported	4 week	**(a)** Mean weight change, kg: +0.6^b^
				**(b)** Mean weight change, kg: +0.6 No significant difference between (a) and (b)

^a^data are mean values (SD).

^b^range not reported.

The duration of the interventions varied from eight to 10 weeks and all were delivered in the community [19,20]. It was unclear whether those delivering the intervention were trained, with one study [19] reporting that the intervention was delivered by a researcher and a recreational therapist but Fisher [20] did not provide such information.

### Intervention components

#### Behaviour change

The behaviour change techniques used in both studies were based on the Rotatori and Fox programme [18]. However, one study [19] eliminated some of the common behavioural strategies including leaving food on plate after eating and conversion techniques of negative reinforcement to diminish cravings. In addition, the paper incorporated an illustration of the resources used (food record chart and the “eating habit” record) attached to the publication [19]. Parents were involved the same ways as in Fox et al. [15] supporting participants with the daily homework and to provide encouragement and reinforcing the main messages of the intervention. No parental involvement was reported in Fisher [20].

Only Fox et al. [19] included a weight maintenance phase of five weeks that immediately followed the 10 week weight loss phase. The weight maintenance phase included meetings where behavioural strategies were reviewed, reinforcement techniques were continued but the daily activity of homework completion was used less intensively. Participants were still encouraged to lose more weight.

#### Physical activity

Fox et al. [19] aimed to increase the energy expenditure of the participants by instructing them to perform calisthenics and aerobic exercises twice a day without specifying the duration. On the other hand Fisher [20] focused on walking exercise introducing 10 minutes of walk at the beginning of the intervention, increased to 30 minutes of walk by the end.

### Study outcomes

Fox et al. [19] showed that the combination of physical activity and behavioural approaches could lead to weight loss greater than 5% at 10 weeks post intervention. However, Fisher et al. [20] showed that incorporation of physical activity had no effect on weight loss.

Fox et al. [19] also assessed the influence of “buddy reinforcement” in the process of weight loss phase reporting inconsistent contacts and no meaningful relationship was established with assigned partners. Therefore, “buddy reinforcement” had no effect on weight loss in this study.

### Study limitations

Once more Fox et al. [19] and in Fisher [20] did not report power calculations and recruited small sample sizes (ranging from 16–17 participants). Both studies reported a random allocation to one of the two intervention groups studied but did not describe the process of random allocation. Contrary to Fisher [20], Fox et al. [19] included a 52 week follow up reporting a mean weight change of −0.6 kg from baseline. No incidents of attrition were reported in both studies.

### Dietary interventions

Dietary interventions all aimed to achieve weight loss with modification to the type, quantity and/or frequency of food and drink consumed to achieve and maintain a hypocaloric energy intake [9]. The interventions did not report using behaviour change strategies or advising on appropriate physical activity interventions to assist weight loss (see Table 3) [21,22].

**Table 3:** Dietary interventions

**Study / Location/ Type**	**Participants**	**Intervention**	**Follow up**	**Results**
Antal 1988 [21] Hungary Institution Uncontrolled quasi-experimental study	Total n = 92 inpatients, recruited: 15 Weight status: All obese Gender: 10 females, 5 males Age (years)^a^: females: 38 (13), males: 44 (15) ID: mainly imbeciles and one Down syndromeAttrition/drop out: none	**Duration:** 9 months **Diet:** 30 day rotating menu of 1000 to 1100 kcal energy content, containing 125 g carbohydrate. Quantity of food was measured once a week. **Maintenance:** none reported	9 months	**Females:** Mean weight change, kg (SD): -16 (2.7) ^b^Mean BMI change, Kg/m^2^: -12.2 **Males:** Mean weight change, kg (SD): -13 (4.5)Mean BMI change, Kg/m^2^: -6.7
Bertoli 2008 [22] Italy Community Uncontrolled quasi-experimental study	Total n = 37 Gender: 12 females, 25 males Age (years) ^a^: 33.5 (9.2) Weight status: 6 obese/overweight ID: 13 with ID (9 Down syndrome, 4 cerebral palsy), the rest were only physically disabled Drop out: 65%, 24 participants (9 of which with ID)	**Duration:** 12 months of individual nutritional counseling led by doctor and dietician (60 min per session). Phone call consultations (15 min) every 3 months.**Diet:** Personalised dietary protocols based on healthy low fat eating and on LARN recommendations. Parents/ legal tutors of ID participants were asked to support participants e.g. dietary changes and completion of food diaries.**Maintenance:** none reported	12 months	For the 6 obese/ overweight participants at baseline: Mean weight change, kg (SD): -6.8 (4) (*p* = 0.01) ^b^Mean BMI change, kg/m^2^ (SD): -2.4 (1.4) (*p* = 0.008) Significant reduction in fat mass (*p* = 0.008) No clarification if the 6 participants had ID.

^a^data are mean values (SD).

^b^range not reported.

The intervention in Antal et al. [21] took place in an institution but no information was reported regarding the profession of the people who delivered the intervention. Bertoli et al. [22] reported that the intervention was delivered by a medical practitioner and a dietitian in the community.

### Intervention components

#### Diet

Antal et al. [21] offered a low calorie diet (1000 to 1100 kcal) to 15 participants with ID and obesity for nine months in the form of a 30 day rotating menu in an institutional setting. There was no further description of the content of the diet. Bertoli et al. [22] offered one to one nutritional counseling to the participants for 12 months in the form of a personalised dietary plan based on their body composition, biochemical parameters and food intake. The plan was focused on the principles of a healthy balanced diet, a reduction in saturated fat and cholesterol intake and based on Recommended Assumption Level of Energy and Nutrients (LARN) for Italian Population. Parents and tutors of participants with ID were asked to assist with food recording and facilitate change in dietary habits.

### Study outcomes

Antal et al. [21] reported a very high weight loss at nine months (mean weight loss:-13 kg for males and -16 kg for females). Bertoli et al. [22] reported on the six participants who were classified as obese or overweight a statistically significant decrease in weight (−6.8 kg) and BMI (p < 0.05) at 12 months. However, there was no clarification of whether these individuals had an ID or not.

### Study limitations

Neither of the studies used power calculations or randomization. The sample size ranged from 15 to 37 participants with Antal et al. [21] offering the intervention to a heterogeneous sample of participants with physical disabilities or ID and Bertoli et al. [22], recruiting only six participants who were obese/overweight out of 37. The study did not investigate the impact of these factors on the results. All participants completed the intervention in Antal et al. [21] but Bertoli et al. [22] had a high dropout rate of 65%. The drop out was mainly attributed to lack of social support.

### Physical activity interventions

Physical activity interventions provided specific exercise programmes and reported weight or BMI changes. The interventions did not report incorporating behaviour therapy or dietary advice to the participants (see Table 4) [23-26].

**Table 4:** Physical activity interventions

**Study/ Location/ Type**	**Participants**	**Intervention**	**Follow up**	**Results**
Rimmer 2004 [23] USA Community Quasi-experimental study with a control group	**(a) Exercise group** n = 30 Weight status: 13% normal, 23%overweight, 64% obese Gender: 53% females, 47% males Age (years)^a^: 38.6 (6.2) ID: all Down syndrome	**Duration:** 12 weeks of 3 exercise group sessions per week (45 min each) led by physiologists and assistants. **(a) Activity:** 30 to 45 min of cardiovascular exercise and 15 to 20 min of muscular strength and endurance.**Maintenance:** none reported	12 weeks	**(a)** Mean weight change, kg: -1^b^
	**(b) Control group** n = 22 Weight status: 14% normal weight, 9% overweight, 77% obese Gender: 59% females, 41% malesAge (years)^a^: 40.6 (6.5) ID: all Down syndromeAttrition/drop out: none			**(b)** Mean weight change, kg: + 1.7 Significant between group difference (*p* < 0.01)
Moss 2009 [24] South Africa Community Uncontrolled quasi-experimental study	Total n = 100Weight status, BMI^a^: 29.3 (6.8) for females, 29 (8.5) for males Gender: 53 females, 47 males Age (years)^a^: 37.1 (10.1) for females, 39.2 (8.9) for males ID: Intellectually aged between 4-12 yr oldAttrition/drop out: none reported	**Duration:** 12 weeks of 3 days per week exercise group sessions.**Activity:** 20 min walking the first 4 weeks and completing 30 min of walking the final 4 weeks.**Maintenance:** none reported	3 months	Females: Mean BMI change, kg/m^2^: -2.74^b^ Males: Mean BMI change, kg/m^2^: -3.1
Wu 2010 [25] Taiwan Institution Uncontrolled quasi-experimental study	Total n = 146 weight status: 31% obese, 16.9% overweight, 45.8% normal weight, 6.3% underweight Gender: Age (years): 19-67 ID: 3.4% mild, 30.8%, moderate, 33.6% severe, 32.2% profound Attrition/drop out: none	**Duration:** 6 months of 4 times per week exercise group sessions (40 min each) led by institutional caregivers.**Activity:** Exercise sessions included sports acrobatics, jogging, dancing, and walking.**Maintenance:** none reported	6 months	Mean weight change, kg: -1.86 (*p* < 0.001)^b^ Mean BMI change, kg/m^2^: - 0.84 (*p* < 0.001)
Mendonca 2011 [26] Portugal Community Quasi-experimental study with a comparison group	**(a) Down Syndrome** n = 13 Weight status, BMI^a^: 29.3 (3.7)Gender: 3 females, 10 malesAge (years): 27-50 ID: Down syndrome, mild -moderate ID	**Duration:** 12 weeks of 3 days per week exercise group sessions led by physiologist and assistants. **Activity:** 2 days combined training separated by one day of endurance training (30 min): treadmill walking or running, dynamic exercises: leg press, chest press, vertical traction, shoulder press, lower back, leg extension, biceps curl, and triceps pushdown, abdominal curls **Maintenance:** none reported	12 weeks	**(a)** Mean BMI change, kg/m^2^:-0.4^b^
	**(b) No Down syndrome** n = 12 Weight status, BMI^a^: 26.6 (4.5)Gender: 3 females, 9 malesAge (years): 27-50 ID: No IDAttrition/drop out: none			**(b)** Mean BMI change, kg/m^2^: 0No significant difference between (a) and (b)

^a^data are mean values (SD).

^b^range not reported.

The majority of the studies (n = 3), with the exception of Wu et al. [25] provided a 12 week intervention. The interventions were delivered in group sessions by physiologists [23,26] or carers [24]. Wu et al. [25] was the only study delivering a physical activity intervention that took place in a disability institution and not in the community.

### Intervention components

#### Physical activity

Physical activity interventions aimed to reduce cardiovascular risk factors, to improve physical fitness and muscular strength of adults with ID. For example Rimmer et al. [23] developed a programme that incorporated regular cardiovascular exercise and activities to improve muscular strength and endurance and Mendonca et al. [26] assessed the effect of aerobic and resistance exercise in exercise economy and peak exercise capacity in adults with Down syndrome.

None of the physical activity interventions included the prescription of 225-300 min or more of moderate intensity physical activity per week to facilitate weight loss, as recommended again by clinical guidelines [8,9]. A total of 135-minutes per week were included in the physical fitness programme in one study [23] and a total of 160-minutes per week by Wu et al. [25]. The type of activities varied and included sports, acrobatics, jogging, dancing or walking [24,25], treadmill walking and circuit exercises [23,26].

### Study outcomes

None of these studies reported weight loss equal or greater than 5% or 5 kg. However, Wu et al. [25] reported a statistically significant decrease in weight and BMI (p < 0.001) at six months.

Despite the minimal effects of physical activity on the weight of the participants, the studies reported positive effects on the cardiovascular fitness, muscular strength, endurance [23], significant decrease in percentage of body fat (−8.0%) and decrease of physical inactivity by 50% [24] and improvement of walking economy (p < 0.05) [26].

### Study limitations

The sample size in the physical activity interventions ranged from 25 to 146 participants. The samples of all of the studies and especially Wu et al. [25] suffered from heterogeneity in relation to the nutritional status and the level of ID of the participants. For example the sample of Wu et al. [25] included 45.8% of participants of normal weight and 6.3% of underweight participants, with levels of ID ranging from mild to profound. Similarly Rimmer et al. [23] recruited participants with normal weight and obesity but all diagnosed with Down syndrome. Mendonca et al. [26] reduced heterogeneity in the sample even more by recruiting participants with Down syndrome and only mild to moderate levels of ID.

Rimmer et al. [23] used power calculations to determine sample size and was the only study that reported using random allocation. However, the random allocation was not adequately described. None of these studies provided follow up assessments of outcome measures. Mendonca et al. [26] identified that lack of blinded assessors for the collection of the pre and post data could act as one of the limitations of the study.

### Dietary plus physical activity interventions

This section includes interventions that provided advice to the participants on how to change their diet and physical activity but did not report using behaviour change techniques to promote the changes (see Table 5)[27-30].

**Table 5:** Dietary plus physical activity

**Study/ Location/ Type**	**Participants**	**Intervention**	**Follow up**	**Results**
Marshall 2003 [27] UK Community Uncontrolled quasi-experimental study	Total n = 25 Weight status: 12% obese, 32% very obese, 36% overweight, 20% normal weight and underweight Gender: 68% males, 32% females Age (years): 30-39 (60%), 12% in their 40s, 12% in their 50s, 12% >60 ID: Down’s syndrome (32%)Attrition/drop out: one	**Duration:** 6 weekly group sessions (2 hr each) led by nurses. **Diet:** healthy eating. **Activity:** advice to be active **Maintenance:** none reported	6 weeks	(n = 20 Overweight and obese participants) Mean weight change, kg: -3.4 (*p* < 0.001) ^b^Mean BMI change, kg/m^2^:-1.6
Bradley 2005 [28]UKCommunityUncontrolled quasi-experimental study	Total n = 9 Weight status: 8 out of 9 obese Gender: all females Age (years): over 18 ID: not reported Attrition/drop out: none	**Duration:** 12 months of 34 group sessions (90 min to 2 hr each) led by a dietitian. **Diet:** information on healthy balanced diet. Food preparation and supermarket visits included. **Activity:** insufficient information.**Maintenance:** none reported	12 months	(n = 7) Mean weight change, kg: -6.2 (range: 2.2 to -15.5)Mean BMI change, kg/m^2^: -3
Chapman 2005, 2008 [29,30] UK Community Quasi-experimental study with a comparison group	Gender: 43% women, 57% men**(a) Intervention group:** n = 38 Weight status: 97% obese and overweight|Age (years)^a^: 37.13 (8.75) ID: not reportedAttrition rate (1-6 years): 13% **(b) No intervention group:** n = 50 Weight status: 64% obese and overweight Age (years)^a^: 43.32 (10.97) Attrition rate (1-6 years): 13% for (a), 20% for (b).	**(a)** Individual sessions led by physiotherapist. **Diet:** advice (no details reported). **Activity:** designed activity programme. Carers were involved in the improvement of lifestyle.**Maintenance:** none reported**(b)** No input	6 months	**(a)** Mean BMI change, kg/m^2^: -0.32^b^
				**(b)** Mean BMI change, kg/m^2^: +0.35, (*p* < 0.05)
			12 months	**(a)** Mean weight change, kg: -1.52^b^ Mean BMI change, kg/m^2^: -0.61 (*p* < 0.05) 42% reached > 1.6 kg weight loss
				**(b)** Mean BMI change, kg/m^2^:+0.41 (*p* < 0.05)
			6 years	**(a)** (n = 40), Mean BMI change, kg/m^2^:-1.02, Mean weight change, kg: -2.42 (range: -28.13 to 14.49, SD 9.15).
				**(b)** (n = 33), Mean BMI change, kg/m^2^: +0.16Mean weight change, kg: +0.61 (range: -18.62 to 16.37, SD 8.81)

^a^data are mean values (SD).

^b^range not reported.

The duration of the interventions varied from six weeks to 12 months and were all led by health professionals e.g. nurses [27], dietitians [28] or physiotherapists [29]. The interventions were community based and mainly delivered in group sessions with one exception [29].

### Intervention components

#### Diet

Three studies that reported providing advice or information in diet and physical activity could be also classified as health promotion or health education interventions [27-30]. Bradley [28] and Marshall et al. [27] used educational material covering healthy eating as part of the content. Specifically, Marshall et al. [27] used an adapted content from the “activate materials” produced by the health promotion agency in Northern Ireland, designed to improve healthy eating and exercise patterns.

#### Physical activity

Both studies did not report sufficient information regarding the advice given on physical activity. Chapman et al. [29] developed activity plans in conjunction with support staff and relatives and offered advice on diet but with insufficient description of the information provided.

### Study outcomes

Bradley [28] reported a weight loss greater than 5 kg at 12 months but omitted any description of statistical analysis used. Marshall et al.[27] reported a significant weight loss (p < 0.001) for the obese and overweight participants (20 out of 25) at six weeks and Chapman et al.[29] did not report weight changes but reported significant decrease in BMI at six months. After a six year follow up the mean BMI decreased by 1.02 kg/m^2^ but not significantly for the group that received the intervention and mean BMI increased by 0.16 for the group that did not receive an intervention [30].

## Study limitations

The sample size in the dietary and physical activity interventions ranged from nine to 25. No power calculations or randomization procedure were reported in any of these studies. A major limitation of all three studies was the insufficient description of the intervention components reducing their reproducibility. Similar to other studies in adults with ID, samples were heterogeneous with Marshall et al. [27] recruiting obese, overweight and normal weight participants and Chapman et al. [30], recruiting mainly obese and overweight participants (97%) but failing to report their level of ID.

All participants completed the intervention of Bradley [28], while one person dropped out of Marshall et al. [27] and reason was not reported. Sixteen people were excluded from the data analysis of Chapman et al. [29] due to lack of data measurement times or due to extreme weight changes not attributed to the intervention.

Chapman et al. [30] included follow up measurements at six years, with a 13% attrition reported for the intervention group and 20% for the usual care group. The study provided a detailed explanation of the attrition, mainly attributed to death or relocation.

### Multi-component interventions

The studies in this section are multi-component interventions defined as a “a combination of diet and physical activity with a behaviour change strategy to influence lifestyle” [14] (see Table 6) [31-38].

**Table 6:** Multi-component interventions

**Study/ Location/ Type**	**Participants**	**Intervention**	**Follow up**	**Results**
Jackson 1982 [31] Australia Community Quasi-experimental study with a control group	Gender: all females **(a)Treatment group** n = 6 Weight status: 10% overweight Age (years), mean: 21.8ID, mean IQ: 38.17 **(b) Control group** n = 6 Age (years), mean: 23.5 ID, mean IQ :40.33 Attrition/drop out: none	**Duration:** 14 weeks of every 2 weeks group sessions (60 min each) led by a teacher. **(a)** 7 sessions with the parents, 6 sessions with group members and the teacher. **Diet:** Advice on healthy eating diet, avoid fad diets. **Activity:** General advice on physical activity e.g. using stairs instead of elevator. **Behaviour:** self-monitoring, reward, punishment, change of rate of eating, reinforcement. **Maintenance**: none reported **(b)** No intervention	17 weeks	**(a)** Mean weight change, kg: -5.75^b^
				**(b)** Mean weight change, kg:-0.59
			3 month	**(a)** Mean weight change, kg :-6.25
				**(b)** Mean weight change, kg: -0.59
			6 month	**(a)** Mean weight change, kg: -6.08
				**(b)** Mean weight change, kg: +0.33
			12 month	**(a)** Mean weight change, kg: -7.33
				**(b)** Mean weight change, kg: 0.00 Significant weight reduction of (a) across all the follow up
Harris 1984 [32] USA Community Quasi-experimental study with a comparison group	Total n = 21 Weight status: not reported **(a) Completers** n = 10 Gender: 8 females, 2 males Age (years)^a^: 22.7 (6.37) ID, IQ^a^: 52.5 (12.80) **(b) Non completers:** 11 Attrition/drop out: 11	**Duration:** 7 weekly group sessions and 1 hour booster session 26 weeks after the first session. **(a) Diet:** education on healthy balanced diet, distinguishing high and low calorie foods, diabetic exchange diet (ADA, 1977). **Activity:** 5-10 min aerobic exercise at the end of session. **Behaviour:** stimulus control, self monitoring, self-reinforcement, goal setting, self-contacting. Carers attended the sessions. **Maintenance:** none reported	7 week	**(a)** Mean weight change, kg: -3.0 (*p* < 0.05)^b^
			12 months	**(a)** Mean weight change, kg: -0.76
				**(b)** Mean weight change, kg: +2.39 (*p* < 0.05) (*p* < 0.05)
Ewing 2004 [33] USA Community Quasi-experimental study with a comparison group	**(a) participants with ID** Total n = 154, final n = 92 Weight status, BMI^a^: 35.4 (7.0) Gender: 54.4% females Age (years)^a^: 39.7 (11.5) ID, IQ^a^: 50.2 (14.3) Attrition/drop out: 18.8% **(b) no ID** Total n = 270, final n = 97 Weight status, BMI^a^: 38.4 (8.6) Gender: 84.5% females Age (years)^a^: 49.9 (11.48) Attrition/drop out: 30%	**Duration:** 8 week intervention. The “HELP” intervention (Health Education Learning Program) led by health educators. 8 group sessions and 2 to 4 home visits. **Diet:** a home visit to develop dietary plan and do a grocery visit. **Activity:** a home visit to develop an exercise programme e.g. walking routes, optional brisk walk after the sessions. **Behaviour:** motivation to change, relapse prevention, avoidance of “automatic thinking”. **Maintenance**: none reported	2 months	**(a)** Mean BMI change, kg/m^2^: 0^b^
				**(b)** Mean BMI change, kg/m^2^: -0.89 No significant difference between (a) and (b)

^a^data are mean values (SD).

^b^range not reported.

The majority of the multi-component weight loss interventions (n = 6) were delivered in group sessions with the exception of the two most recent studies that offered individual interventions [37,38]. Other studies like Ewing et al. [33] and Mann et al. [34] offered a home visit to develop an individualized physical activity programme and a dietary plan in addition to the group sessions.

The qualifications of those who delivered the interventions varied but included health professionals specialized in ID e.g. health educators [33], physicians [36], and dieticians [37,38].

### Intervention components

#### Diet

Two studies included energy deficit diets as part of the intervention [37,38]. Melville et al. [37] recommended dietary change based on a personalised dietary prescription that was calculated to achieve an energy deficit of 600 Kcal (2510 kJ) per day and a weight loss of 0.5 kg to 1 kg/week. Saunders et al. [38] recommended a low calorie diet of 1200 to 1300 kcal (5024 to 5442 kJ) per day focusing on the consumption of high volume foods that provide the sensation of fullness (Volumetrics). The dietary intervention also included meal-replacement drinks providing 110 calories per serving and a “Stoplight Guide” classifying food into three coloured categories: green for less than 60 calories, yellow for 60 to 100 calories and red for over 100 calories [38].

Other studies that included a dietary change component were two studies that offered home visits to the participants to develop individualized dietary plans [33,34]. One study provided dietary information based on the Diabetic Exchange Diet [32]. The rest of the studies provided limited information about the nutritional advice that was offered to the participants. These studies mainly took the form of health education programmes providing general information regarding healthy dietary habits and patterns e.g. healthy meal planning [31,35,36]. Cooking classes, meal planning and grocery store visits were common activities relevant to diet among the interventions [34-36,38].

#### Physical activity

None of the multi-component studies provided an exercise programme that promoted 225-300 min or more of moderate intensity physical activity per week [8,9]. Five of the studies incorporated physical activity programmes (sometimes optional) as part of the intervention sessions, offering dancing, aerobic exercises and walking [32,34-36,38].

Jackson and Thorbecke [31] provided advice to make simple lifestyle changes e.g. taking the stairs instead of the lift and Melville et al. [37] recommended that participants work towards 30 minutes of moderate intensity physical activity, on at least five days per week.

As part of the intervention Melville et al. [37] used a specially designed DVD aiming to motivate participants to become more active while Geller and Crowley [36] used an exercise video. Both resources included only people with ID. In addition, Melville et al. [37] provided participants with information regarding local leisure centers that they could attend. Pedometers were also used to motivate participants to be more active through walking [37,38].

#### Behaviour change

The behaviour change techniques that were used as part of the multi-component interventions included goal setting, strategies to improve motivation, problem solving, stimulus control and relapse prevention strategies [32-34,37]. Geller and Crowley [36] mainly focused on empowering the participants by enhancing their ability to make choices and by creating feelings of community and success in groups. Self-monitoring was facilitated with weight and food diaries [31,37,38] and reward systems were used to motivate behavioural change [31,35,37].

Harris and Bloom [32] and Bazzano et al. [35] invited the main carers of all participants to be present during the sessions of the weight loss intervention. However, no description of their role was reported in the study. Saunders et al. [38] asked carers to assist participants when they appeared to be having difficulties to respond to specific questions. Jackson and Thorbecke [31] described a similar role for the parents to Fox et al. [15,19] but parents were also instructed to deliver punishment statements when participants ate “prohibited foods” or withdraw a reward if weight increased. Melville et al. [37] also invited the carers to be present at the sessions, assisting the consultation where appropriate, encouraging the participants during the weight loss process.

Saunders et al. [38] was the only multi-component intervention that recommended to participants at the conclusion of the dietary intervention ways of increasing calorie intake to prevent further weight loss. This was followed by a six month less intensive phase, involving monthly meetings but discontinuing the request that participants complete food and exercise records, stopping the supply of low calorie shakes and the incentive rewarding.

### Study outcomes

All of the multi-component interventions reported a decrease in weight, or BMI but it appears that the greatest weight loss was that reported by the two interventions that recommended energy deficit diets [37,38]. At six months follow up Saunders et al. [38] reported a 6.3% weight loss from baseline and Melville et al. [37] reported a mean weight loss of 4.3%. Melville et al. [37] reported that 36% of the participants reached a 5% weight loss. An intervention that involved the parents of the participants intensively reported a 6.07% weight loss at week 17 and a total weight loss of 10.36% from baseline, at 12 months [31]. The weight loss (5.75 kg) in adults with ID that completed the intervention was significantly different from the controls (0.59 kg) that were not offered an intervention (p < 0.05).

### Study limitations

The majority of the studies of multi-component weight loss interventions recruited small numbers of participants, none were based on pre-treatment sample size estimations (n = 12-192), and included obese and overweight participants, based on BMI scores. Only one study limited inclusion criteria to participants with obesity [37]. No power calculations and no randomization were used by any of the multi-component studies.

Two studies reported weight changes at follow up at least 12 months from baseline [31,32]. All of the multi-component studies reported attrition or dropout rates with the highest attrition rate up to 35% [35]. Bazzano et al. [35] reported that barriers to attendance included lack of motivation to exercise, transportation, childcare, conflicting work schedules, and language translation needs. Ewing et al. [33] showed that when home visits were added to the analysis of attendance at more than four classes of the intervention, attendance was higher among the group with home visits (87%) compared with those without a home visit (79%).

## Discussion

Similar to other reviews [3-6], a limited number of studies in lifestyle weight management for adults with ID and obesity were found. In general, over the years people with ID and obesity have had a minimal involvement in research [39] despite expressing their interest to participate [40]. There is no research examining the specific reasons of exclusion of individuals for ID from weight management studies. However, this can be explained by the already identified challenges in developing research for adults with ID, especially in relation to ethics. Several studies and ethics committees have looked at the ethical issues related to the types of interventions provided to people with ID, reporting the necessity of interventions tailored to the needs of the participants and reviewing the principles and procedures that need to be followed when individuals with ID have not the capacity to consent their participation in a study [41].

### What components are included in weight loss interventions for adults with ID?

Inconsistency in the methodology of the studies and insufficient information regarding the components of the interventions used made their classification into a specific category difficult. The physical activity and behaviour change components of the interventions were more clearly described in most of the studies in comparison with the dietary aspects of the interventions. This limitation can affect the reproducibility of the studies and has been also identified in weight management studies for adults without ID [14].

Several clinical guidelines recommend that obesity management interventions should use a multi-component model that incorporates advice on dietary behaviour and physical activity patterns [7-9,14] and should also include behaviour change techniques to help individuals achieve sustainable changes in these lifestyle areas [7-9,14]. However, few studies (n = 8) were classified as multi-component interventions in this review.

A 600 kcal energy deficit is identified as a realistic amount of energy deficit that can lead to a loss of adipose tissue and sustained weight loss of 0.5 kg per week, ensuring a better compliance from individuals with obesity [42,43]. However, very few studies in this review used energy deficit diets with Melville et al. [37] being the only study that offered a 600 kcal energy deficit diet to the participants. The absence of studies examining the effectiveness of energy deficit diets in this population group may be related to the challenging issues that may arise implementing a significant change in the routine of an individual with ID, especially when the individual has autism [44]. It is possible that researchers and carers may consider that a healthy balanced diet will not disturb the dietary patterns of an individual with ID to a great extent and will not cause distress. However, a 600 kcal energy deficit diet can be based on the same principles as a healthy balanced diet requiring small changes for a small sustained weight loss. This issue has not been investigated by other studies or reviews but a qualitative investigation on the opinions and beliefs of researchers and carers could provide an insight into this.

The benefit of physical activity in the management of obesity depends on the amount and the intensity of the intervention [45,46]. Clinical guidelines for the treatment of obesity recommend more than 225-300 min per week of moderate intensity physical activity [8,9]. None of the studies provided an exercise programme that followed these recommendations. However, this amount of exercise may not be realistic for adults with ID, a population group with a very sedentary behavior [47] and resistant to change daily routines [48]. This means that adults with ID may require longer periods to reach and sustain this amount of daily physical activity than adults without ID.

Behaviour change techniques in weight management aim to support and maintain changes in cognitive behaviour in relation to eating habits or activity patterns of individuals with obesity [8]. Most common behaviour change techniques used in studies for adults with ID in this review are the same with those identified in interventions for adults without ID: self-monitoring, goal setting, reward strategies and relapse prevention [13,49-51]. However, contrary to the behaviour change techniques used in weight management interventions for adults without ID [14], the intervention for adults with ID did not state if they were based on a specific theory (e.g. stages of change of the Transtheoretical model of change or the Social Cognitive theory).

Several studies in this review reported that carers were involved at different levels with poor description of their role and with only three of them describing the impact of their involvement on weight loss [15,16,31]. Willner et al. [52] reported that carers can have a vital role in motivating individuals with ID in the process of cognitive therapy and readiness to change. This finding was supported by Spanos et al. [53] that explored in depth the role and the experiences of the paid and family carers that participated in Melville et al. [37] According to the qualitative study the carers provide encouragement and praise to the participants in a weight loss intervention and assist in the process of goal setting, essential mechanisms for behaviour change in obesity management.

The majority of the interventions were delivered in group sessions, which could be regarded as more preferable potentially offering improved cost effectiveness [54]. However, there is insufficient evidence to support the effectiveness of group therapy for weight management versus individual therapy [50,55]. No studies in this review explored or commented on which method is the most suitable way of delivering a weight loss intervention for adults with ID.

To reduce health inequities that adults with ID frequently experience while using health services [56] weight loss interventions should be made accessible by tailoring the intervention to the cognitive, communication and literacy abilities of adults with ID [57]. Some of the reviewed studies highlighted the importance of developing an intervention based on the needs of the people with ID by describing the resources and the adaptations that had to be followed [19,27,35,37].

### Are weight loss interventions for adults with ID associated with a clinically significant weight loss?

Even though there were studies that did not report robust statistical analysis, the majority of the studies reported weight loss based on weight or BMI. Some studies reported changes in waist circumference [28,32,35,37,38] or waist hip ratio [22,24] but the results are not reported in this review.

According to clinical guidelines for obesity and weight management, for individuals with BMI 25–35 kg/m^2^ with no comorbidities present a 5-10% weight loss (approximately 5-10 kg) is required for the reduction of obesity related health risks [7-9]. Three studies reported a clinically significant weight loss within six months: one behaviour change and physical activity intervention [19], and two multi-component interventions [31,38]. Other studies reported a clinically significant weight loss at nine months [21] and at 12 months [22,28]. Limitations and the differences in methodology and intervention components do not allow comparisons or support of the effectiveness of these studies. However, the absence of use of energy deficit diets and the lack of recommended levels for physical activity, may partly explain the poor weight loss outcomes in these studies.

This review focused only on first line treatment of obesity and did not examine pharmacotherapy and surgery in adults with ID, treatments that could potentially be effective in this population group. However, to our knowledge no studies have examined the effectiveness of this type of weight management in adults with ID and obesity and this can be explained by the ethical issues related with such type of weight management for this population.

### Do interventions include a weight loss maintenance component?

Weight loss maintenance following a weight loss intervention is important, showing that individuals who have lost weight and maintained their weight have made sustainable lifestyle changes that will prevent future weight gain or health risks [8,9]. However, research for weight management in the general population has mainly focused on the development and evaluation of weight loss strategies and has not examined extensively the effectiveness of weight maintenance interventions that follow a weight loss phase [58]. Only four studies out of the 22 in this review offered a structured weight loss maintenance intervention [15,16,19,38], with weight loss being still promoted in two of these studies [15,19].

### Methodological limitations

A major limitation of this literature is the absence of sample justifications making it likely that these studies are under powered given the small sample sizes (ranging from 6 to 192). A review of 20 studies in this population group showed that lack of direct contact when inviting individuals with ID to participate in a study, inclusion of invasive procedures such as blood testing and the procedures of taking consent may discourage poor participation in the studies for adults with ID [59].

Only two studies recruited participants from institutional settings [21,25] and the rest from community settings. Samples were usually heterogeneous, especially in relation to the level of ID. Level of ID was reported in different ways including as mean IQ scores [33] or percentage of mild, moderate and profound ID [37] or not reported [28]. In addition, some studies used strict inclusion criteria and offered an intervention only to participants that had mild to moderate ID and others offered an intervention to participants with a variety of levels of severity of ID. This may have had an impact on the level of support from the carers leading the studies to making inappropriate generalisations of the effectiveness of their intervention.

The same pattern of sample heterogeneity was also seen in relation to the weight status of the participants. For example Melville et al. [37] delivered a multi-component weight loss intervention to obese participants only but Chapman et al. [29] offered a diet and physical activity intervention to a group of participants who ranged from a healthy weight to the overweight or obese and it was even more surprisingly that Wu et al. [25] included normal weight and underweight participants in their study. Most of the studies provided the same intensity of intervention to participants that were obese, overweight and sometimes normal weight. According to clinical guidelines [8,9] the intensity of a dietary intervention (600 kcal energy deficit) can be the same for overweight and obese individuals but the intensity of the physical activity intervention and the targets of weight loss may need to change based on the BMI and the associated health risks of their weight to an individual.

Only four studies reported using randomised allocation [19,20,23,31]. Allocation concealment to the intervention or control groups was unclear for all these studies. RCTs are regarded as the most “powerful tool” in research, especially for the evaluation of healthcare interventions [59]. However, it is essential for these studies to explain the process of random allocation because a detailed description ensures that these studies are truly randomized aiming to reduce the limits for bias [10,60]. For example, studies that report being randomized but not reporting using a method of concealment and have allocated participants by using the date of birth (odd and even numbers) are not regarded as randomized [10].

There was no consistency in the duration of the interventions varying from two months to 12 months. According to a recent clinical guideline [9] most individuals are able to lose weight actively for about three to six months and so studies reporting ‘weight loss’ at 12 months actually measure a mixture of weight loss and weight maintenance.

According to the clinical guidelines [7-9], the effectiveness of weight loss interventions is also associated with the duration that the weight loss is maintained. This aspect of weight management can be evaluated with long term follow up measurements after the intervention. However, the longest follow up measurements reported in this review were by Chapman et al. [30] at six years followed by one study reporting measurements at 18 months [36] and four at 12 months [16,19,31,32].

High attrition levels are common incident among weight loss interventions, with a usual attrition rate range of 30%-60% [61]. Attrition is used to judge the acceptability of interventions, as it often reflects participants’ high weight loss expectations and low initial weight loss [62]. The majority of the studies included in this review did not report a high dropout or attrition rate with the exception of one dietary intervention and a multi-component intervention [22,38].

### Limitations of the review

One of the great difficulties in the review of weight management interventions is the classification of an intervention to a category (e.g. multi-component, physical and dietary interventions) but also to provide a description of their components (e.g. behaviour change). The process can be seen as quite biased and subjective and it has been seen in other reviews where different or unclear definitions have been used, especially in the case of the multi-component interventions. However, this review described and evaluated the components of each intervention using the specific recommendations of national and international guidelines, a method that has not be used in other reviews of this area of research.

## Conclusion

Overall the studies that assessed weight loss in adults with ID suffer from similar limitations in sample, design and analysis leading to insufficient evidence to support the effectiveness of a particular intervention. This systematic review has highlighted the need for future weight management interventions in adults with ID that will be based on the recommendations from national clinical guidelines on the use of multi-component interventions, including “user friendly” resources, ensuring and defining the pivotal role of carers and offering a structured weight loss maintenance phase as part of a multi-component weight loss intervention.

## Abbreviations

ID: Intellectual disabilities; BMI: Body mass index; RCT: Randomised controlled trial; kJ: Kilojoules; Kcal: Kilocalories; LARN: Recommended assumption level of energy and nutrients.

## Competing interests

All co-authors have seen and agree with the contents of the manuscript and there is no financial interest to report. We certify that the submission is original work and is not under review at any other publication.

## Authors’ contributions

DS conceived of the study, and participated in its design, carried out the review and drafted the manuscript. CM participated in the design of the study and helped to draft the manuscript. CH participated in the design of the study and its coordination and helped to draft the manuscript. All authors read and approved the final manuscript.
