# Erratum to: weight management interventions in adults with intellectual disabilities and obesity: a systematic review of the evidence

**DOI:** 10.1186/1475-2891-13-123

**Published:** 2014-12-22

**Authors:** Dimitrios Spanos, Craig Andrew Melville, Catherine Ruth Hankey

**Affiliations:** College of Medical, Veterinary and Medical and Life Sciences, University of Glasgow, Glasgow, G31 2ER UK; Learning Disabilities Psychiatry, College of Medical Veterinary and Life Sciences, Institute of Mental Health & Wellbeing, University of Glasgow, Glasgow, G12 0XH UK; Human Nutrition, College of Medical, Veterinary and Medical and Life Sciences, University of Glasgow, Glasgow Royal Infirmary, Level 2, New Lister Building, Glasgow, G31 2ER UK

## Correction

Following the publication of this article [1], we noted errors to Table six (Table 1 here). Corrected version is presented below.

**Table 1:** **Multi-component interventions**

Study/ Location/ Type	Participants	Intervention	Follow up	Results
Jackson 1982 [31]	Gender: all females	**Duration:** 14 weeks of every 2 weeks group sessions (60 min each) led by a teacher.	17 weeks	**(a)** Mean weight change, kg: -5.75^b^
				**(b)** Mean weight change, kg:-0.59
Australia	**(a) Treatment group**	**(a)** 7 sessions with the parents, 6 sessions with group members and the teacher. **Diet:** Advice on healthy eating diet, avoid fad diets. **Activity:** General advice on physical activity e.g. using stairs instead of elevator. **Behaviour:** self-monitoring, reward, punishment, change of rate of eating, reinforcement.	3 month	**(a)** Mean weight change, kg :-6.25
				**(b)** Mean weight change, kg :-0.59
			6 month	**(a)** Mean weight change, kg: -6.08
				**(b)** Mean weight change, kg: +0.33
Community	n = 6	**Maintenance**: none reported	12 month	**(a)** Mean weight change, kg: -7.33
Quasi-experimental study with a control group	Weight status: 10 % overweight	**(b)** No intervention		**(b)** Mean weight change, kg: 0.00
	Age (years), mean: 21.8			Significant weight reduction of (a) across all the follow up
	ID, mean IQ : 38.17			
	**(b) Control group**			
	n = 6			
	Age (years), mean: 23.5			
	ID, mean IQ :40.33			
	Attrition/drop out: none			
Harris 1984 [32]	Total n = 21	**Duration:** 7 weekly group sessions and 1 hour booster session 26 weeks after the first session.	7 week	**(a)** Mean weight change, kg:-3.0 (*p* < 0.05) ^b^
USA	Weight status: not reported	**(a) Diet:** education on healthy balanced diet, distinguishing high and low calorie foods, diabetic exchange diet (ADA, 1977). **Activity:** 5–10 min aerobic exercise at the end of session. **Behaviour:** stimulus control, self-monitoring, self-reinforcement, goal setting, self-contacting. Carers attended the sessions.	12 months	**(a)** Mean weight change, kg :-0.76
Community	**(a) Completers**	**Maintenance:** none reported		**(b)** Mean weight change, kg :+2.39 (*p* < 0.05)
Quasi-experimental study with a comparison group	n = 10			(*p* < 0.05)
	Gender: 8 females, 2 males			
	Age (years) ^a^: 22.7(6.37)			
	ID, IQ ^a^ : 52.5 (12.80)			
	**(b) Non completers:** 11			
	Attrition/drop out: 11			
Ewing 2004 [33]	**(a) participants with ID**	**Duration:** 8 week intervention. The “HELP” intervention (Health Education Learning Program) led by health educators. 8 group sessions and 2 to 4 home visits.	2 months	**(a)** Mean BMI change, kg/m^2^ : 0 ^b^
USA	Total n = 154, final n = 92	**Diet:** a home visit to develop dietary plan and do a grocery visit. **Activity:** a home visit to develop an exercise programme e.g. walking routes, optional brisk walk after the sessions. **Behaviour:** motivation to change, relapse prevention, avoidance of “automatic thinking”.		**(b)** Mean BMI change, kg/m^2^: -0.89
Community	Weight status, BMI^a^: 35.4 (7.0)	**Maintenance**: none reported		No significant difference between (a) and (b)
Quasi-experimental study with a comparison group	Gender: 54.4 % females			
	Age (years) ^a^ : 39.7 (11.5)			
	ID, IQ^a^ : 50.2 (14.3)			
	Attrition/drop out: 18.8 %			
	**(b) no ID**			
	Total n = 270, final n = 97			
	Weight status, BMI^a^:38.4 (8.6)			
	Gender: 84.5 % females			
	Age (years) ^a^:49.9 (11.48)			
	Attrition/drop out: 30 %			
**Study/Location/Type**	**Participants**	**Intervention**	**Follow up**	**Results**
Mann 2006 [34]	Total n = 324, available data for 192	**Duration:** 8 weekly group sessions (90 min each) using health education “Steps to Your Health” led by trained staff . Included home visits.	9 weeks	Mean BMI change, kg/m^2^: -0.31^b^ (*p* < 0.05)
USA	Weight status, BMI^a^ : 35.38 (6.85)	**Diet:** individual dietary plan and a grocery store visit. **Activity:** optional brisk walking and individual exercise programme. **Behaviour:** motivation to change, relapse prevention, barriers to change.		
Community	Gender:66.7 % females, 33.3 % males	**Maintenance:** none reported		
Uncontrolled quasi-experimental study	Age (years) ^a^ : 38.6 (11.5)			
	ID, IQ^a^ : 50.7 (13.3)			
	Attrition/drop out: 20 %			
Bazzano 2009 [35]	Total n = 85 signed up, 44 completers	**Duration:** 7 months of 2 weekly group sessions (120 min each) led by professionals specialized in ID. “The Healthy Lifestyle Programme” focusing on health education and peer mentoring. Phone calls included.	7 months	Mean weight change, kg:-1.2 (*p* < 0.05) ^b^
USA	Weight status: 36.4 % overweight, 38.6 % obese, 18.2 % very obese	**Diet:** education and cooking demonstration. **Activity:** education and supervised physical activity (90 min). Exercise in local parks and fitness facilities. **Behaviour:** behaviour modification and reward systems. Carers were encouraged to attend the sessions.		Mean BMI change, kg/m^2^: -0.5 *(p* < 0.05) ^b^
Community	Gender: 61 % females, 39 % males	**Maintenance:** none reported		
Uncontrolled quasi-experimental study	Age (years): 18-59			
	ID: 68 % mental retardation, 25 % ≈ mental retardation, Cerebral palsy, epilepsy, and autism diagnosed in 15 % to 20 %			
	Attrition/ drop out: 35 %			
Geller 2009 [36]	Total n = 45	**(a):** Empowerment model. 1st year: Twice weekly sessions led by a physician (60 min each): Group and individual sessions based on the “Funk” model. **Diet:** meal planning, cooking demonstrations. **Activities:** music chairs, dancing, exercise to music. **Behaviour:** Activities creating feelings of community, feelings of success and of being important.	2 months	**(a) + (b)** (n = 43) Mean weight change, kg:-0.26^b^
			6 months	**(a) + (b)** (n = 38) Mean weight change, kg:-0.78
			12 months	**(a) + (b)** (n = 36) Mean weight change, kg:-0.74
			18 months	**(a) + (b)** (n = 14) Mean weight change, kg:-2.73
USA	Gender: 25 females, 18 males	**(b):** 2nd year, once weekly group sessions -same as (a)		
Community	Weight status: obese/ overweight:-Age (years): average 42.6	**Weight maintenance:** none reported		
Uncontrolled quasi-experimental study	ID: not reported			
	Attrition/ drop out: 2 dropped out, 14 completed 18 month measurements			
Melville 2011 [37]	Total n = 54	**Duration:** 6 months of 9 sessions every 2–3 weeks individual consultations (45-60 min each) based on the GCWMS led by a dietician and a medical graduate.	6 months	Mean weight change, kg (SD): -4.47 (4.45) (*p* < 0.0001) ^b^
UK	Weight status, BMI^a^ : 40 (8.03)	**Diet:** 600 kcal/d energy deficit diet. **Activity:** aim for 30 min of moderate physical activity for 5 days per week. **Behaviour:** goal setting, problem solving, cue avoidance, stimulus control. Carers were encouraged to assist if needed.		Mean BMI change, Kg/m^2^:-1.82 (*p* < 0.0001)
Community	Gender:40.7 % males, 59.3 % females	**Maintenance:** none reported		
Uncontrolled quasi-experimental study	Age (years)^a^:48.3 (12.01)			
	ID: 31.5 % mild, 31.5 % moderate, 35.2 % severe, 1.9 % profound.			
	Attrition/drop out: 3 dropped out, 4 non completers on time			
**Study/ Location/ Type**	**Participants**	**Intervention**	**Follow up**	**Results**
Saunders 2011[38]	Total n = 79 registered, Weight status: mean BMI 38.0	**Duration:** 6 months of one individual session (60-90 min) and monthly consultations (30 min each) led by dietician, behaviour analysts, physiologists.	6 months	(n = 73) Mean weight change, kg:-6 ^b^
USA	Gender: 41 % males, 59 % females	**Diet:** a 1200 to 1300 kcal/d diet based on volumetrics, at least 5 portions of fruits and vegetables; up to three low-calorie, meal/snack-replacement shakes; two packaged entrees of less than 300 calories each and other low calorie items, 2 shake mixes from Health Management Resources (HMR) daily (110 kcal per serving). **Activity:** Optional. A game board aiming to increase number of steps. **Behaviour:** praise, problem solving, reward system. Carers could assist if needed.		Mean BMI change kg/m^2^ ≈ -2.7 % mean weight loss: 6.3
Community	(6 month completers)	**Maintenance:** 6 months of less intensive meetings. Weight loss could continue if wanted.		
Uncontrolled quasi-experimental study	ID: not reported			
	Attrition/drop out: 73 six month completers, 43 twelve month completers			

^a^ data are mean values (SD).

^b^range not reported.
